# A deep multi-stream model for robust prediction of left ventricular ejection fraction in 2D echocardiography

**DOI:** 10.1038/s41598-024-52480-y

**Published:** 2024-01-24

**Authors:** Jennifer Alvén, Eva Hagberg, David Hagerman, Richard Petersen, Ola Hjelmgren

**Affiliations:** 1 Department of Electrical Engineering, Chalmers University of Technology, Gothenburg, Sweden; 2 Department of Molecular and Clinical Medicine, Institute of Medicine, Sahlgrenska Academy, University of Gothenburg, Gothenburg, Sweden; 3 Department of Clinical Physiology, Sahlgrenska University Hospital, Region Västra Götaland, Gothenburg, Sweden; 4 Pediatric Heart Centre, Queen Silvia Children’s Hospital, Sahlgrenska University Hospital, Region Västra Götaland, Gothenburg, Sweden

**Keywords:** Biomedical engineering, Echocardiography, Computer science

## Abstract

We propose a deep multi-stream model for left ventricular ejection fraction (LVEF) prediction in 2D echocardiographic (2DE) examinations. We use four standard 2DE views as model input, which are automatically selected from the full 2DE examination. The LVEF prediction model processes eight streams of data (images + optical flow) and consists of convolutional neural networks terminated with transformer layers. The model is made robust to missing, misclassified and duplicate views via pre-training, sampling strategies and parameter sharing. The model is trained and evaluated on an existing clinical dataset (12,648 unique examinations) with varying properties in terms of quality, examining physician, and ultrasound system. We report $R^2 = 0.84$ and mean absolute error = 4.0% points for the test set. When evaluated on two public benchmarks, the model performs on par or better than all previous attempts on fully automatic LVEF prediction. Code and trained models are available on a public project repository.

## Introduction

Echocardiography is one of the most common, versatile and cost-effective imaging technique for cardiovascular evaluation^1^. Estimation of LVEF (left ventricle ejection fraction) is an important part of the assessment of systolic function. It is defined as the percentage of the left ventricle end diastolic volume that is ejected with each contraction and is often calculated with biplane Simpson method^2^, but also relies on visual assessment “eyeballing” ^3^. However, evaluation of LVEF with echocardiography is associated with uncertainty because of interobserver variation, with better reproducibility among experienced readers^3^. Deep learning methods for 2DE analysis can help towards a more automated, consistent and accurate assessment process^4^.

We propose a deep model for LVEF prediction in 2DE examinations based on an 8-stream convolutional neural network (CNN) and transformer model. We use four 2DE views as input: apical two-, three- and four-chamber (A2C, A3C, A4C), and parasternal long axis (PLAX), which are automatically selected from the full 2DE examination. We focus on 2DE datasets with properties that are common in clinical settings: with varying quality, examining physician and 2DE system, with limited metadata such as missing view information, and with missing or duplicate views. The model is made robust to missing, misclassified and duplicate views via customised pre-training, sampling strategies and parameter sharing. The model is trained, validated and tested on an existing clinical dataset, and in addition, evaluated on two public benchmarks: the EchoNet-Dynamic dataset^5^, and the CAMUS dataset^6^. The methods and the results are reported in accordance with the PRIME checklist, see the Supplementary Table S1.^7^.

There have been several previous attempts to determine LVEF from 2DE examinations with deep models. Some models segment the cardiac chambers in one or several 2DE views, and compute the LVEF from these segmentations^6,8–10^. Others determine LVEF directly from the 2DE examination without an intermediate segmentation step^11–16^, or use a mix of the two approaches^5^. Most often, LVEF determination is posed as a prediction problem^5,6,8–10,12–15^,while some pose it as a classification problem^11,16^. A majority use datasets with known view labels^5,6,8,11,13–16^, while only a few address the more challenging problem with unknown views^9,10,12^. Some only use the A4C view as input^5,11,13,15,16^, others use both A2C and A4C^6,8,9,12^, and a few use the five views A2C, A3C, A4C, PLAX and parasternal short axis (PSAX)^10,14^. Some evaluate their LVEF determination model on publicly available datasets: the EchoNet-Dynamic dataset^5,11,15^, and the CAMUS dataset^6,8,9^.

## Methods

### Model

**Figure 1:**
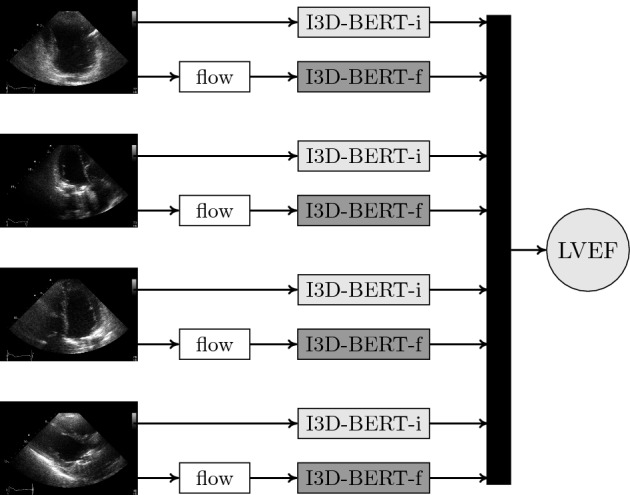
The proposed 8-stream model. Videos (image + optical flow) from four 2DE views are used as input, which are analysed by the image-processing model, and the flow-processing model. The output of the eight streams are combined in a linear layer.

We use four 2DE views as input for the LVEF prediction model: A2C, A3C, A4C and PLAX. These views were selected since they are standard views normally included in most 2DE examinations, and since they contain information that should be helpful for LVEF determination. We use a standard classification model to extract the selected views from the full 2DE examination detailed in the section on pre-processing. For LVEF prediction, we use a 2-stream CNN and transformer model with four views (image + optical flow) as input, that is, eight streams of data in total. See Fig. 1 for a graphical summary of the full model.

The base building block of our LVEF prediction model is the 2-stream I3D model in Carreira and Zissserman, which consists of two 3D CNNs, one for the image and one for the optical flow of the image^17^. Our reasoning behind using a model originally intended for action recognition is the common denominator of having spatiotemporal data. We modify each 3D CNN by adding a terminating BERT (Bidirectional Encoder Representations from Transformers) layer for temporal pooling, which showed improved results on action recognition tasks in Kalfaoglu et al.^18^. The motivation for using a terminating BERT layer is to fully exploit the temporal information using transformer attention mechanisms without loosing any information due to averaging and ignored ordering. We use the FRMB (feature reduction by modified block) solution in Kalfaoglu et al. for this^18^. The same instance of the model (i.e. shared weights) is used to process all the views. We let the four views share parameters since (i) we want the model to be robust to missing, misclassified and duplicate views, and (ii) 2DE views share many common features which allows for a more compact model. The outputs from the eight streams of data are combined in a linear layer. Since each examination might include none, one or several videos of each view class, we construct input data instances according to the following rules: (i) If a view is missing, it is replaced with another view according to the following (descending) priority order: A4C, A2C, A3C, PLAX. This is possible due to the model’s shared weights between views, and should increase the model’s robustness to missing or misclassified views. (ii) If an examination includes several instances of the same view, we create data instances of all possible view combinations. This should increase the model’s robustness to examinations with varying quality, and works as an augmentation strategy. Further, it eliminates the need for a more sophisticated view classifier that chooses between videos of the same view. Details on the optical flow computations can be found in the section on pre-processing.

### Data

The study is a retrospective register study. Inclusion criteria were: (i) A 2DE performed at the Department of Clinical Physiology, Sahlgrenska University Hospital, Gothenburg, Sweden between 2007 and 2017, (ii) 2DE performed on a GE ultrasound system (GE Vivid 7, GE Vivid 9, GE Vivid e9), (iii) clinical report signed by an experienced physician with more than 500 signed reports, (iv) saved image data with minimum one of the following views: A2C, A3C, A4C or PLAX, and (v) a numeric value of LVEF in the clinical report. No exclusions were made due to quality issues (reverberations, artefacts, noise). We included all examinations that fulfilled the inclusion criteria and where LVEF was reduced. We balanced the dataset by adding a randomized sample of examinations with normal LVEF. The final dataset consists of 2DE examinations from 12,648 unique patients where LVEF is reduced in 50% of the examinations, supranormal in 1.5%, and normal in 48.5%. Normal LVEF is defined according to Lang et al. as $\ge 52\%$ for men and $\ge 54\%$ for women^19^. Supranormal is defined as $\ge 70\%$, which is the definition of supranormal used at the site. Since examinations above 70% were classified as 70% (see below), we could not use the definition by Lang et al. (men 72%, women 74%).

**Figure 2:**
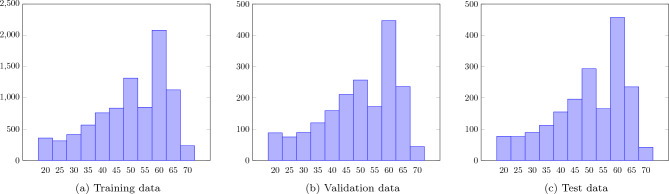
The distribution of LVEF (%) for the (**a**) training data, (**b**) validation data, and (**c**) test data.

Each examination contains 2DE video(s) from one or several views, and corresponding metadata such as heart rate (HR), frames-per-second (FPS), and LVEF. The dataset is split into a training set (70%, 8853 examinations), a validation set (15%, 1898 examinations) and a test set (15%, 1897 examinations). The examinations do not include view metadata, and a view classifier was used to generate view labels for all videos. All examinations include at least one video classified as any of the included views (A2C, A3C, A4C, PLAX). The ground truth LVEF values were manually reported at the time of examination by the examining physician, either by “eyeballing” or by calculations with biplane Simpson or Teichholz method^20^. The videos differ in size, length and FPS. All LVEF values were at the time of examination reported in multiples of five. LVEF values below 20% were reported as 20%, and LVEF values above 70% were reported as 70%. Note that this procedure of binning and truncation of the LVEF values is the standard practice of the echocardiography lab where the dataset was generated. The study population is summarized in Table 1, and the distribution of LVEF values are reported in Fig. 2. All data was anonymized before use and informed consent was not obtained from the study subjects. This protocol was approved, and the need for informed consent was waived, by the Clinical Medical Research Ethics Board of Sweden (ref. number: 818-18). The study was performed in accordance with this ethical approval and the Declaration of Helsinki. The dataset is described in detail in Hagberg et al.^21^.

**Table 1:** A summary of the study population.

	All subjects	Training set	Validation set	Test set
	$n=12648$	$n=8853$	$n=1898$	$n=1897$
Age (years)	67 (56–77)	67 (56–77)	68 (57–78)	67 (56–76)
Female sex	38%	38%	39%	35%
Heart rate (bpm)	72 (62–85)	72 (62–85)	73 (62–85)	72 (62–85)
LVEF (%)	50 (40–60)	50 (40–60)	50 (40–60)	50 (40–60)
Weight (kg)	78 (68–90)	78 (68–90)	78 (67–89)	79 (68–90)
	$n=11869$	$n=8338$	$n=1771$	$n=1760$
Length (cm)	173 (165–180)	173 (165–180)	172 (165–180)	174 (166–180)
	$n=11866$	$n=8345$	$n=1766$	$n=1755$
LVDd (mm)	51 (47–56)	51 (47–56)	51 (47–56)	51 (47–56)
	$n=10938$	$n=7678$	$n=1648$	$n=1612$
LVEF assessed with				
Eyeballing	74.5%	74.5%	75.1%	74.0%
Biplane Simpson	24.5%	24.4%	24.2%	25.3%
Teichholtz	0.9%	1.1%	0.7%	0.7%

The data is presented as median (interquartile range) and/or counts *n*.

*LVDd* left ventricle diastolic diameter.

### Pre-processing

Videos were converted from RGB to grayscale via averaging, and pixels values were normalized to $[-1, 1]$. Frames were resized to the same size ($223\times 169$ pixels), and the videos were resampled to have the same FPS/HR, set to 18 frames/heartbeat. This temporal resolution was carefully selected to strike a balance between minimizing computational complexity (favoring lower temporal resolution) and preserving video interpretability (favoring higher temporal resolution). To guarantee that each input video includes one complete cardiac cycle, we used the first 20 frames for each resampled video (corresponding to $\approx 1.1$ cardiac cycles), and resampled videos with less than 20 frames were periodically extended (“looped”) before resampling. A subset of pre-processed videos was inspected by experienced physicians to make sure that it was possible to analyse LVEF with this pre-processing, and that all inspected videos contained a full heart cycle with both end systolic and end (or near end) diastolic information. We used the Dual TV L1 optical flow algorithm in Zach et al.^22^. implemented in OpenCV 4.4.0, for flow computations (stopping criterion threshold 0.05, number of warpings per scale 1, number of pyramid scales 1). This is the same optical flow algorithm used by Carreira and Zisserman^17^, and Kalfaoglu et al.^18^.

We used a pre-trained Inception2D (v3) from Szegedy et al. for view classification^23^, where the first convolutional layer was modified to have one input channel (grayscale) instead of three (RGB) by averaging the pre-trained weights. We used five different view classes: (i) A2C, (ii) A3C, (iii) A4C, (iv) PLAX, and (iv) other (which includes all other views). The classes A2C, A3C and A4C include views with optimized depth settings for focusing on the left ventricle. We annotated the view labels for a subset of the examinations in the dataset, and divided these into a training set (70%, 381 examinations), a validation set (10%, 55 examinations) and a test set (20%, 103 examinations). The model was trained on predicting the views from individual frames, the view class for a full video was computed with majority voting. We used the AdamW optimizer^24^, learning rate (LR) 1e−4, batch size 8, weight decay 2e−5 and the cross-entropy loss (weighted with respect to class distribution). The model reached an average accuracy of 94% for the test set, with class accuracies 95% (A2C), 98% (A3C), 93% (A4C), and 95% (PLAX). Additional view classification networks were also explored, see Hagberg et al., while the best performance was obtained using Inception2D (v3), which is why this architecture was selected^21^.

### Training

We used the training set for learning the weights of the LVEF prediction model, and the validation set for hyperparameter and model selection. The LVEF model was trained with a 3-step procedure: (i) I3D without BERT was pre-trained on ImageNet, we re-used the weights in Kalfaoglu et al.^18^. (ii) 2-stream I3D with BERT was trained on mixed views, and (iii) 8-stream I3D with BERT was trained on separated views. We used same the training strategies, and the same set of hyperparameters, for step (ii) and (iii). The training data was sampled with respect to continuously updated sample weights computed from the loss for each training data instance. The weight for each training instance was set to the ratio between the training instance loss and the corresponding batch loss, and clamped to [0.1, 3] to avoid extreme sample weights. If an examination in the training set included duplicate views, we sampled one of the possible inputs with an uniform probability over all possible inputs. We did not use any other augmentation strategies since evaluated techniques (e.g. using different types of noise, geometric transformations, brightness adjustments, occlusion strategies) gave no significant improvements on the validation set. We used the AdamW optimizer^24^, LR 1e−3, batch size 16, weight decay 1e−4, loss function MSE, half-precision accuracy for input data and a LR scheduler that decreases the LR by a factor 1e1 if no improvement on the validation loss can be seen after 100 iterations (validation loss were computed every 10th iteration). If an examination in the validation set included duplicate views, we used weighting when computing the evaluation metrics such that each examination had a total weight equal to one. For all experiments, we used PyTorch 1.7.1 with Cuda 10.1. Average training time was 136 h (500 iterations) on a Nvidia DGX-2 with 22 CPUs and four V100 GPUs, with a memory footprint of 218 GB RAM and 19 GB per GPU.

### Evaluation

We evaluated the model’s performance on an internal test set with 1897 examinations, on two public benchmark datasets and in an ablation study investigating the impact of (i) sharing the weights between the models processing each view, (ii) replacing missing views with other view classes according to the predefined priority order, (iii) using all available view instances in case of duplicate views, (iv) using optical flow as a second data stream, (v) using BERT as a terminating layer, (vi) different loss functions and (vii) different number of input views. We used bias ± standard deviation (SD), the coefficient of determination $R^2$ and the mean absolute error (MAE) as evaluation metrics.

## Results

### Test set results

**Figure 3:**
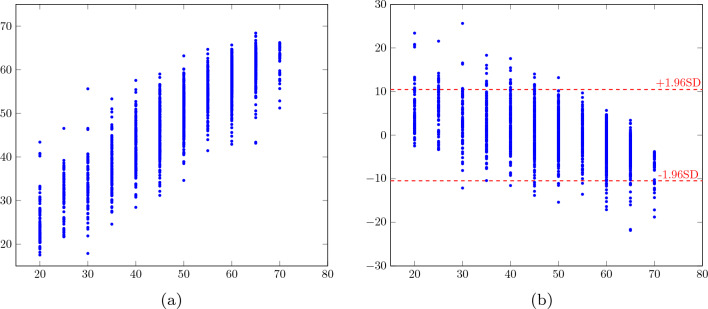
(**a**) Scatter plot with with predicted LVEF % versus target LVEF (%). (**b**) Bland-Altman plot with difference between predicted and target LVEF (pp) versus target LVEF (%). Red dashed lines = ± 1.96SD.

We used the 1897 examinations in the test set for testing the model’s final performance. If an examination in the test set included duplicate views, we used averaging over all possible outputs to compute the LVEF prediction. See Table 2 for bias ± SD, $R^2$ and MAE depending on the available input views, and Fig. 3 for scatter and Bland-Altman plots.

**Table 2:** Performance of the proposed 8-stream model depending on the available views for all test examinations with all views available, pp = percentage points.

N/A views	Bias ± SD (pp)	MAE (pp)	$R^2$
–	0.1 ± 5.2	4.0	0.84
PLAX	− 0.5 ± 6.7	5.5	0.73
A4C	− 0.7 ± 5.9	4.8	0.79
A3C	− 0.6 ± 6.3	5.2	0.76
A2C	− 0.8 ± 6.1	5.0	0.77

### CAMUS and EchoNet-dynamic results

Table 3 reports the model’s performance when evaluated on the CAMUS and the EchoNet-Dynamic test datasets, as well as previously reported LVEF prediction results for these datasets.

For the CAMUS dataset, we finetuned and evaluated a 4-stream version of the model, since the CAMUS dataset only includes A2C and A4C views. We used the training dataset for finetuning (450 examinations) and the test dataset for testing (50 examinations). We employed the model trained on our in-house dataset for initialization, and used the identical learning strategy and hyperparameters as outlined in the Training section. The CAMUS examinations only include the contraction phase (systole), and lack heart rate metadata. Therefore, we resampled all videos to have 20 frames corresponding to one contraction.

For the EchoNet-Dynamic dataset, we finetuned and evaluated a 2-stream version of the model, since the dataset only includes A4C views. We used the training dataset for finetuning (10751 examinations) and the test dataset for evaluation (1897 examinations). Since the EchoNet-Dynamic examinations lack heart rate metadata, we resampled all videos to have 20 frames with 9 frames/second. As for the CAMUS dataset, we used the model trained on our in-house dataset for initialization, and the same learning strategy and hyperparameters as outlined in the Training section.

**Table 3:** Performance of the proposed model, and previous models, for LVEF prediction for the CAMUS and the EchoNet-Dynamic datasets, pp = percentage points.

Method	Dataset	Bias ± SD (pp)	MAE (pp)	$R^2$
Ours	CAMUS	− 0.1 ± 2.4	1.9	0.94
Leclerc et al.^6^	CAMUS	0.5 ± 7.7	5.6	0.79
Liu et al.^8^	CAMUS	− 1.7 ± 4.1	n/a	n/a
Smistad et al.^9^	CAMUS	1.8 ± 8.9	6.7	n/a
Ours	EchoNet-Dynamic	− 0.3 ± 5.4	4.1	0.81
Ouyang et al.^5^	EchoNet-Dynamic	n/a	4.1	0.81
Reynaud et al. ^15^	EchoNet-Dynamic	n/a	6.0	0.52

### Ablation study

Table 4 reports $R^2$ and MAE values for the following comparisons on the validation set: (i) w/o shared model weights between the different views, (ii) w/o view replacement in case of missing views by using empty views instead, (iii) w/o using all available views in case of duplicate views, (iv) w/o optical flow as a second data stream in the I3D models, (v) w/o BERT as a terminating layer in the I3D models, (vi) with MSE (regression), balanced all-threshold (ordinal regression) and balanced cross-entropy (classification) as loss function, and (vii) with one input view (A4C) in a 2-stream model, two input views (A2C, A4C) in a 4-stream model, three input views (A2C, A3C, A4C) in a 6-stream model, and four input views (A2C, A3C, A4C, PLAX) in an 8-stream model. To ensure a fair comparison, the LVEF predictions are rounded to the nearest multiple of five for the MSE loss in comparison (vi), and the validation set only includes examinations with at least one A4C (1870 examinations) in comparison (vii).

**Table 4:** $R^2$ and MAE (pp = percentage points) values for the validation set for the proposed model versus (i) w/o shared model weights between the different views, (ii) w/o view replacement, (iii) w/o duplicate views, (iv) w/o optical flow, (v) w/o BERT, (vi) balanced all-threshold (ordinal regression) and balanced cross-entropy (classification) loss, and (vii) with 1, 2, 3 input view(s).

		$R^2$	MAE (pp)
Proposed model		0.83	4.12
w/o	Shared weights	0.82	4.13
w/o	View replacement	0.81	4.28
w/o	Duplicate views	0.77	4.69
w/o	Optical flow	0.82	4.13
w/o	BERT	0.65	6.48
Loss	MSE	0.81	4.13
Loss	All-threshold loss	0.81	4.17
Loss	Cross-entropy loss	0.80	4.26
Views	1	0.78	4.53
Views	2	0.79	4.45
Views	3	0.81	4.25
Views	4	0.82	4.21

Note that the first model (“Proposed model”) and the last model (views: 4) are the same models evaluated on different validation sets the first one is evaluated on the full validation set while the last one is evaluated only on examinations with at least one A4C view (to ensure a fair comparison to 1/2/3 views).

## Discussion

In this paper, we present a fully automatic model for LVEF assessment, with a MAE of 4.0 pp. We conclude from Table 2 that while depending on all included views for optimal performance, the model relies less on A2C, A3C and A4C than on PLAX, which is expected due the somewhat overlapping information (from the same apical window) in A2C, A3C, and A4C compared to PLAX using a different ultrasound window. One possible reason for the less impressive performance for LVEF = 20% and 70% than the intermediate values in Fig. 3 is the truncation of the ground truth labels, which may have resulted in a model prone to overestimate small LVEF values, and correspondingly, to underestimate large LVEF values.

Second, we conclude that using BERT as a terminating layer is crucial for optimal performance, which is expected due to transformers’ superior capability to incorporate temporal information compared to e.g. average or max pooling. Further, we conclude that using all possible view instances in case of duplicate views boosts the model’s performance, which is of little surprise since it increases the amount of available training data. Further, the model’s performance consistently improves with an increased number of views, and using optical flow seems to boost the performance somewhat. However, these improvements (more views, and multiple data streams) need to be weighted against the increased computational complexity they add. Using view replacement gives a modest performance boost, however, it has the advantage of adding no computational complexity. Similarly, using shared weights between the different views only gives a small performance boost, while it has the advantage of significantly reducing the computational complexity. Surprisingly, there is only small differences between posing LVEF determination as a regression, ordinal regression and classification problem.

When comparing the proposed model to previous methods on the CAMUS dataset, we can conclude that we outperform all previous works in terms of bias, MAE, $R^2$ and SD. When comparing the proposed model to previous methods on the EchoNet-Dynamic dataset, we can conclude that we outperform one, and perform on par with another, in terms of MAE and $R^2$. Our dataset comprises real-world clinical examinations from hospital archives, with no exclusions based on image quality. We view this as a strength, as more curated datasets, such as CAMUS, tend to present a simplified problem. Conducting a prospective test in a clinical setting would undoubtedly provide a valuable opportunity to further assess performance.

We envision (at least) three immediate research directions. Firstly, when evaluating, the video most suitable for LVEF determination should be automatically selected when there are duplicate views, instead of using weighting/averaging. Secondly, the output from each stream should be paired with a learned model confidence to enable a more sophisticated fusion of the output from each stream. Finally, the model should be implemented in a picture archiving and communication system, and evaluated in a clinical setting, including analysis of the clinical significance, usability and reliability.

## Limitations

Our paper has several limitations. (i) One limitation arises from the nature of our dataset, a real-world clinical dataset. Specifically, LVEF values in this dataset have been truncated, with values below 20% set to 20% and values exceeding 70% set to 70%. While this truncation aligns with clinical practices, it introduces a potential drawback in our study. It may lead to an overestimation of low LVEF values and an underestimation of high LVEF values. It’s important to acknowledge, however, that the clinical relevance of this limitation is likely minimal. (ii) Another limitation stems from the fact that we did not explore various methods for balancing the dataset in favor of impaired LVEF values. Employing such balancing techniques could have potentially improved our results, particularly for low LVEF values. (iii) A further limitation pertains to the handling of duplicate views during examinations. In our analysis, we incorporated all possible inputs from duplicate views and averaged the LVEF measurements obtained from them. This approach deviates from a more refined dataset, where only the best-case duplicate view is selected. However, it is essential to note that our analysis is fully automated, and incorporating a manual step to choose the optimal duplicate view is not a practical option. In summary, while our study is not devoid of limitations, we believe that these constraints do not significantly detract from the clinical implications of our findings.

## Conclusions

We have proposed a deep 8-stream model for LVEF prediction in 2DE examinations using four automatically selected 2DE views as input. The model was trained and evaluated on an existing clinical dataset with varying quality, examining physician and 2DE system, with limited metadata such as missing view information, and with missing or duplicate views. We reported $R^2 = 0.84$, MAE = 4.0% points and bias = 0.1 ± 5.2% points for the test set. We also evaluated on two public benchmarks. These datasets differ significantly from our focus: they include only A2C/A4C and A4C views respectively, no examinations have missing or duplicate views, and view labels are known. Still, we performed on par or better than all previous LVEF prediction methods evaluated on these two datasets.

### Supplementary Information

Supplementary Table S1.

## Data Availability

The in-house dataset analysed during the current study are not publicly available since the ethical approval does not allow for this. However, the dataset is available from the corresponding author on reasonable request including an approved ethical approval by the Clinical Medical Research Ethics Board of Sweden. The two benchmark datasets are publicly available at the CAMUS project homepage and EchoNet Dynamic homepage. The trained models are available on a public project repository.
