# Supplementary material for: A deep multi-stream model for robust prediction of left ventricular ejection fraction in 2D echocardiography
Source: Sci Rep. 2024 Jan 24;14:2104. doi: 10.1038/s41598-024-52480-y (PMC10808096; doi:10.1038/s41598-024-52480-y)
Supplement: Supplementary file 1 — Supplementary Table S1. [file 41598_2024_52480_MOESM1_ESM.docx]

| Supplementary Table S1: The PRIME checklist. | | | |
| --- | --- | --- | --- |
| **Section** | **Checklist item** | **Alvén et al.** |  |
| **1** | **Designing the study plan** |  |  |
| 1.1 | Describe the need for the application of machine learning to the dataset | Page 1 |  |
| 1.2 | Describe the objectives of the machine learning analysis | Page 1 |  |
| 1.3 | Define the study plan |  |  |
| 1.4 | Describe the summary statistics of baseline data | Page 3 |  |
| 1.5 | Describe the overall steps of machine learning workflow | Page 3 |  |
| **2** | **Data standardization, feature engineering, and learning** |  |  |
| 2.1 | Describe how the data were processed in order to make it clean, uniform, and consistent | Page 3 |  |
| 2.2 | Describe whether variables were normalized and if so, how this was done | Page 3 |  |
| 2.3 | Provide details on the fraction of missing values (if any) and imputation methods | Page 3 |  |
| 2.4 | Perform and describe feature selection process | Page 3 and 4 |  |
| 2.5 | Identify and describe the process to handle outliers if any | Page 3 |  |
| 2.6 | Describe whether class imbalance existed, and which method was applied to deal with it | Page 3 |  |
| **3** | **Selection of Machine Learning Model** |  |  |
| 3.1 | Explicitly define the goal of the analysis e.g., regression, classification, clustering | Page 3 and 4 |  |
| 3.2 | Identify the proper learning method used (e.g., supervised, reinforcement learning etc.) to address the problem | Supervised |  |
| 3.3 | Provide explicit details on the use of simpler, complex, or ensemble models | Page 2 and Fig. 1 |  |
| 3.4 | Provide the comparison of complex models against simpler models if possible | Page 4 |  |
| 3.5 | Define ensemble methods, if used | - |  |
| 3.6 | Provide details on whether the model is interpretable | Page 4 and 5 |  |
| **4** | **Model Assessment** |  |  |
| 4.1 | Provide a clear description of data used for training, validation, and testing | Page 3 |  |
| 4.2 | Describe how the model parameters were optimized (e.g., optimization technique, number of model parameters etc.) | Page 4 and 5 |  |
| **5** | **Model Evaluation** |  |  |
| 5.1 | Provide the metric(s) used to evaluate the performance of the model | R, R^2^, MAE, MSE |  |
| 5.2 | Define the prevalence of disease and the choice of the scoring rule used | Not applicable |  |
| 5.3 | Report any methods used to balance the numbers of subjects in each class | Page 3 |  |
| 5.4 | Discuss the risk associated to misclassification | Page 5 |  |
| **6** | **Best Practices for Model Replicability** |  |  |
| 6.1 | Consider sharing code or scripts on public repository with appropriate copyright protection steps for further development and non-commercial use | Available on GitHub |  |
| 6.2 | Release data dictionary with appropriate explanation of the variables | Available on the public data repositories |  |
| 6.3 | Document version of all software and external libraries | Available on GitHub |  |
| **7** | **Reporting limitations, biases and alternatives** |  |  |
| 7.1 | Identify and report the relevant model assumptions and findings | Page 2 |  |
| 7.2 | If well performing models were tested on a hold-out validation dataset, detail the data of that validation set with the same rigor as that of training dataset (see section 2 above) | Page 3 |  |
